# Surveillance of Resistance to New Antibiotics in an Era of Limited Treatment Options

**DOI:** 10.3389/fmed.2021.652638

**Published:** 2021-04-19

**Authors:** Chantal M. Morel, Marlieke E. A. de Kraker, Stephan Harbarth, The Enhanced Surveillance Expert Consensus Group (CANSORT-SCI)

**Affiliations:** ^1^University of Geneva Hospitals & Faculty of Medicine, Geneva, Switzerland; ^2^University Hospital Bonn, Institute for Hygiene and Public Health, Bonn, Germany; ^3^Infection Control Programme, University of Geneva Hospitals and Faculty of Medicine, Geneva, Switzerland; ^4^WHO Collaborating Centre on Patient Safety, University of Geneva Hospitals and Faculty of Medicine, Geneva, Switzerland

**Keywords:** resistance surveillance, antibiotics, antimicrobial resistance, antimicrobial susceptibility, early warning systems

## Abstract

As with any health threat, our ability to respond to the emergence and spread of antimicrobial resistance depends on our ability to understand the scale of the problem, magnitude, geographical spread, and trends over time. This is especially true for resistance emergence to newer antibiotics coming to the market as last-resort treatments. Yet current antibiotic surveillance systems are limited to monitoring resistance to commonly prescribed drugs that have been on the market for a long time. This qualitative study determined the essential elements and requirements of antimicrobial resistance surveillance for new antibiotics based on literature review, interviews and expert consensus. After an extensive mapping exercise, 10 experts participated in a modified Delphi consultation to identify consensus on all elements required for surveillance of resistance to novel antibiotics. The main findings indicate that there is a need for a two-phase system; an early alert system transitioning to routine surveillance, led by the public sector to gather and share essential data on resistance to newer antibiotics in a transparent manner. The system should be decentralized, run largely from national level, but be coordinated by an arm of an existing international public health institution. Priority should be given to monitoring emergence of resistance among already multi-drug resistant pathogens causing infections, over a broader selection of pathogens to maximize clinical impact. In conclusion, we cannot rely on current AMR surveillance systems to monitor resistance emergence to new antibiotics. A new, public system should be set-up, starting with a focus on detecting resistance emergence, but expanding to a more comprehensive surveillance as soon as there is regional spread of resistance to the new antibiotic. This article provides a framework based on expert agreement, which could guide future initiatives.

## Introduction

Over the years, antimicrobial resistance (AMR) has been steadily increasing, and new resistance mechanisms have emerged and spread worldwide (1–3). This threatens the effective treatment of patients suffering from community-acquired and healthcare-associated bacterial infections, as well as the success of prophylactic treatment in patients undergoing high-risk surgery. As such, there is a clear need for the development of effective, novel antibiotic treatments (4). While the antibiotic pipeline has been slowly refilling, only one in four new, approved antibiotics represents a novel drug class or mechanism of action, and to-date none of these are active against Gram-negative WHO critical threat pathogens (5). At the same time, the emergence and spread of AMR also requires vigilance to identify changes in the epidemiology of pathogens causing infections—including their resistance to newer antibiotics. Yet, our current surveillance systems focus only on resistance to older drugs. They are not designed to adequately measure resistance to the newer ones used for last-resort treatment.

Current, publicly-led surveillance efforts focus on pathogen resistance within the set of older antibiotics. For example, in Europe, EARS-Net collects data from invasive isolates (blood and cerebrospinal fluid), reporting resistance amongst seven common pathogens^1^ to carbapenems, fluoroquinolones, third-generation cephalosporins, aminoglycosides, and penicillins (6). GLASS also collects data on these pathogen-antibiotic combinations, in addition to resistance to a few more, such as tetracyclines, penicillins, sulfonamides plus trimethoprim—all from the older generation of antibiotics (WHO 2). Occasionally some more targeted measures have been taken in response to specific concerns about resistance to new drugs. For example, the European Center for Disease Prevention and Control (ECDC) recommended notifying cases of resistance to the newly marketed broad-spectrum antibiotic ceftazidime-avibactam and exchanging information through existing platforms^2^ to enable informed and coordinated action by public health authorities (7). For the most part, AMR surveillance for newer drugs has been initiated by the private sector to support applications for (additional) market approval or to satisfy post-launch requirements to the regulator. Some of the more well-known private sector surveillance initiatives include MYSTIC, ResistNet, and the Alexander Project. However, information from these surveillance activities remain the property of companies who collect it, usually through third parties. A selection of findings from these surveillance activities are published occasionally, but often with limited detail regarding the sampling protocol. This makes it difficult to compare with other data, assess potential for reporting or selection bias, and to assess the level of representativeness and overall usefulness for public health policy. These resistance surveillance findings are certainly never sufficiently detailed or thorough to spur urgent public health action. Asking industry (antibiotic producing pharmaceutical companies and the surveillance companies they contract) to be the *de facto* lead on AMR surveillance of newer antibiotics creates a clear conflict of interest as resistance to their product affects efficacy, and, in turn, decreases future sales. This can engender biases in the data published and ultimately further limit its usefulness. For example, in a recent study, it was shown that the ATLAS data, from Pfizer, consistently reported higher resistance proportions for older antibiotics than did public data from EARS-Net (8).

Given the burden to collect surveillance data and the conflicts of interest involved in depending on industry, the European Medicines Agency (EMA) is anticipating reform to lower post-market surveillance monitoring obligations by companies to the European regulator (9), which will lower the amount of data they collect and publish. Private sector surveillance activities have already slowed down, and altogether ceased within some companies. The public sector will be left to fill the gap if we are to have any insight into emerging resistance—and hence efficacy—of our most valuable new, antibiotic treatments. This study explores the requirements and desired features of a standardized system for monitoring bacterial resistance to newly launched antibiotics within the healthcare setting in order to help advance discussions on how best to enhance and improve AMR surveillance of new antimicrobial agents. The reported results are based on a mapping exercise, combined with a consensus process based on input from international experts.

## Materials and Methods

### Issue Mapping

The first phase of this qualitative study consisted of a mapping exercise to explore key features of a surveillance system for novel antibiotics (those with novel mechanisms of action) through a review of literature and interviews (17–46).

The literature review included studies published between 1998 and 2019 in MEDLINE, using the following search criteria: resistance surveillance, antibiotic/antimicrobial resistance, antibiotic/antimicrobial susceptibility, susceptibility testing. In addition, reference lists of relevant studies were screened, and experts were consulted to identify all relevant studies. This informed the various features of AMR surveillance and helped to evaluate previous and ongoing AMR surveillance systems.

Twenty interviews were then undertaken with a range of stakeholders involved in AMR surveillance, including microbiologists, hospital epidemiologists, infectious disease physicians, private sector companies, and specialists from national and international agencies (e.g., ECDC, CDC, WHO) based in Europe and the United States^3^. Experts were identified from a combination of the authors' professional networks, published literature, and snowball sampling (identifying one led to the identification of another). Interviews were intended to move one step beyond the published literature, to gather key aspects of the surveillance structures that need to be considered when designing an enhanced, publicly-led AMR surveillance framework that would cover new antibiotics.

### Consensus Procedure

Findings from the mapping exercise were used to develop a survey and conduct a modified Delphi consultation. Delphi was chosen amongst numerous other potential expert elicitation methods due to its flexibility and usefulness for very specific questions. The limited number of experts ruled out random participant selection. The inability to repeatedly bring all the experts together for face-to-face moderating ruled out other elicitation forms. Delphi is a structured process that uses a series of rounds to gather information from a heterogeneous panel of experts in order to achieve agreement. Agreement of 70% among responses was used as a threshold to define consensus. Respondents remained anonymous to one another and provided their responses in isolation through structured questioning. The software *Survey Monkey* was used to conduct the survey.

Experts, all external to the study, were selected on the basis of their broad methodological expertise in AMR surveillance: microbiology, infectious disease, epidemiology, and public health. They were identified from their publications in the field and/or their involvement with the main surveillance networks (e.g., EARS-Net, GLASS, Combacte-Magnet, EPI-Net). Overall, 11 experts were identified and invited to participate^4^.

Delphi Round 1, which took place in late 2018, consisted of 26 questions covering the key features of a surveillance system: the patient population, the isolate selection process, the sample types, the timing of isolate collection, the institutions that should be reporting to the system, etc. (Appendix 1 in Supplementary Materials). The survey was piloted by three individuals: two microbiologists and one infectious disease specialist. Ten survey responses were received (gender balance: four women, six men) from Europe and North America.

Delphi Round 2, which took place in spring 2019, included the same 10 participants. The survey included the findings from Round 1 followed by 10 additional questions (see Appendix 2 in Supplementary Materials), which sought to gain greater precision on the topics of agreement, to clarify questions or responses from Round 1 that had been deemed ambiguous, and to identify the root of any disagreement. Several of the questions derived from a case study (of a hypothetical “super-penem”), used to understand multidimensional preferences (using scoring 1 to 5). The last question of the questionnaire was posed in an open format to ask participants for any further comments regarding the ideal features of early warning or routine AMR surveillance for new antibiotics. Responses were received from all ten experts who had participated in Round 1.

Expert consultation was terminated when, for each question, there was either broad consensus or consensus was deemed impossible but clarity on the basis for disagreements was achieved, as discussed below.

## Results and Discussion

The first part of the issue mapping exercise, based on a literature review, identified nine main surveillance design issues (focus and purpose, timing, governance, antibiotic susceptibility testing (AST) data, AST methods, type of sampling, pathogen, desired patient-level data, desired hospital-level data, data quality, reporting) with 61 sub-issues (Appendix 3 in Supplementary Materials). The second part, based on expert interviews, narrowed the issues down to those requiring expert consensus for prioritization and a more in-depth exploration through the Delphi exercise (Appendices 1, 2 in Supplementary Materials).

### Focus and Purpose of Resistance Surveillance

The objectives of AMR surveillance can differ across systems. Generally, they are intended to achieve the objects listed in Table 1.

**Table 1:** Objectives of surveillance systems [adapted from (13) and (16)].

Establish the prevalence of different forms of resistance, their geographic distribution, and their evolution over time (including outbreaks)
Inform the estimation of burden
Guide empirical therapy
Inform and monitor prevention activities
Provide data to assess the efficacy/effectiveness of interventions
Detect new resistance mechanisms
Evaluate the threat of transmission of an especially worrying resistance mechanism or clone
Bolster capacity-building, standardization and harmonization of antimicrobial susceptibility testing across laboratories
Detect and report abnormal bacteriological events such as low levels of acquired resistance
Suggest transmission of resistance genes between species
Explore the consequences of bacterial resistance over time, such as relationship to patterns in treatment failure, morbidity, mortality, or economic impact

For AMR surveillance of new antibiotics generally most of the above objectives would qualify as well. However, for novel drugs, since acquired resistance will hopefully be a rare occurrence right after market approval, and laboratory methods for resistance detection might not yet have been developed, it will be more complicated to achieve *all* of the listed objectives.

#### Timing: Two-Stage Surveillance

As described below, the Delphi exercise clarified that enhanced surveillance for new antibiotics should be conducted in two phases; first an early warning surveillance to detect emergence of resistance, followed by an enhanced routine surveillance to determine frequency, spread, setting and trends.

#### Phase 1. Early Warning Surveillance to Detect Emergence of Resistance

Immediate early warning surveillance—to be implemented as soon as a newly introduced drug is used locally—was deemed to be particularly relevant for drugs that are critically important, such as for the treatment of infections with limited treatment options (e.g., multidrug resistant–MDR) and priority AMR threats [e.g., carbapenem-resistant Enterobacterales–CRE, as per (10–12). Such immediate surveillance was considered crucial to act quickly with regard to infection prevention and control measures, and both development and dissemination of diagnostic procedures (including procuring appropriate technologies). Immediate surveillance was considered less important for new members of existing classes that do not substantively add to the existing treatment options. In this study, respondents agreed that the level of use of the new antibiotic was not relevant; while normally all novel antibiotics should have very low levels of use initially, this should not preclude surveillance.

There was some initial disagreement amongst experts regarding the issue of who should be responsible for reporting emergence of resistance to the surveillance governing body. Half of respondents (5/10) in Round 1 felt that, for initial reporting of resistance emergence, we could rely on agreements with existing reference laboratories, while the other half felt we should rely on agreements with existing (largely private) surveillance networks. In Round 2 participants were asked how we should proceed if private sector resistance surveillance activities decrease following the expected EMA regulation reform which greatly decreases the surveillance duties of private companies. Four out of five participants who had initially reported that we should rely on specialized private sector surveillance companies to report resistance emergence ultimately agreed that it would be more prudent to rely on public reference laboratories for this purpose in view of the expected reduction in private sector activity. The one dissenting voice qualified the response, pointing out that many public laboratories in Asia and Africa are inadequate, implying that this may not be a concern in Europe or United States. Acceptance of public reference laboratories for reporting of resistance emergence in Europe and the US was therefore considered unanimous. The reliance on reference laboratories globally may not be currently possible^5^.

All hospitals should be expected to send isolates suspected of resistance to the novel drug (including all repeat isolates of that patient) to a reference lab where the resistance can be confirmed and the evolution of the strain can be explored. The role of the reference lab in detecting early resistance was seen to be active—meaning that the reference lab should receive guidelines and have some form of obligation to report to the surveillance body. Many participants felt that the World Health Organization (WHO) early warning system (EAR)—which is a reporting system, not a surveillance system—could be useful for reporting initial resistance emergence, assuming subsequent verification. In Phase 1 the number of resistant samples and patients should be reported initially, soon followed by reporting of resistance proportions in order to have some indication of the relative importance of the problem. Burden of disease measures, like prevalence or incidence were not seen to be essential for early warning surveillance. (See below for further discussion of resistance measures).

#### Phase 2. Routine Surveillance

All participants agreed that, as resistance becomes more prominent, routine surveillance should be enhanced, and the selection of institutions for routine surveillance should facilitate as good a coverage as possible. Ideally, the results of AST for new antibiotics should be reported for all samples, including pediatric isolates, by all facilities processing clinical samples. Of course, practically this depends on the level of available resources and infrastructure. For example, if it is possible to connect all laboratory information systems (LIS) to a central database, then all available AST results can be automatically uploaded to the surveillance structure.

The transition between the two phases of surveillance was discussed as part of a hypothetical (“super-penem”) case study (see below). In the next paragraphs, we will provide a more detailed description of essential elements of routine AMR surveillance.

### Governance: Structure and Funding of the Enhanced Surveillance System

Availability of resources can clearly influence how any resistance surveillance system is designed and assumptions about the availability of resources affects how experts foresee the enhancement of current surveillance practice. Therefore, the resource question was addressed in many of the themes explored below.

In general, international public AMR surveillance systems have been based on networks of networks, where for example national laboratory networks report to an international network—often without any underlying funding—while private AMR surveillance systems have had a more top-down approach supported by funding. Expert consultation suggested that governance of an enhanced surveillance system should mimic public AMR surveillance systems; be run largely from national level, but coordinated by an arm of an existing international public institution (e.g., WHO, ECDC). The enhanced surveillance would require financial support, as this will affect the degree of authority of the governing structure. For example, EARS-Net currently does not offer contributing countries or hospitals any funding, and therefore cannot impose specific sampling schemes, or apply centralized testing. Funding from the public health sector would safeguard transparency.

### Governance: Data Dissemination Structure

All Delphi participants agreed that AMR surveillance data should be merged at national/sub-national level first and then fed to the international governing body. It was emphasized that curation of AMR data by national level authorities is preferred prior to sharing it with any supranational entity. In particular, from a quality perspective, the first level of data collection, cleaning, validation, and confirmation should be carried out at the national level. The EARS-Net method for centralizing the data is considered a well-functioning, tried and tested model. However, for the purposes of early warning, direct contact between the laboratories and the surveillance body may be required to avoid delays. Timeliness is indeed crucial, and all efforts must be made to avoid lags in cleaning and verification. Withholding information in anticipation of publishing must of course be strongly discouraged.

### Governance: Hospital Selection

Owing to limitations of resources and the focus on new antibiotics intended for use within the hospital setting for multidrug resistant infections, it was assumed that sentinel surveillance (a small number of selected surveillance sites) would be used over wider, population-based surveillance—even if the former reduces external validity and risks missing the first, emergent strains. At the same time, representativeness of the data provided by the selected centers for similar centers in the country should be an explicit goal. (Representativeness is discussed further in the “Type of sampling” section below).

For surveillance sites, participants agreed that local hospitals (clinical records), not local laboratories (microbiological reports), or reference laboratories should be the site of choice to report data to the governing body. Lab-based often means that clinical data is unavailable, which complicates, or renders impossible, the distinction between contamination, colonization, or infection. Surveillance based on clinical records can be syndrome-driven, has the advantage of availability of clinical outcome data, and can provide metrics to indicate potential detection bias, like “Number of blood cultures conducted per 1,000 patient-days within the contributing hospital” (preferred detection bias metric by respondents).

No consensus could be achieved regarding who should be in charge of selecting hospitals for the sentinel surveillance: six participants thought it should be the surveillance governing body, while 4 argued that it should be left to national authorities. Participants also did not agree on whether a country should have to provide data from a representative set of hospitals/laboratories (*N* = 5 votes) or a convenient sample (*N* = 5 votes) in order to be a full member of the surveillance network. From the free text it became clear that this disagreement was not associated with differences in valuing representativeness, but rather the expectancy that some national governments under-value AMR surveillance and will not put the effort into collecting representative data, thereby possibly rendering the most important countries ineligible to participate. Indeed, WHO and ECDC currently go through national governments and public health authorities, and as indicated several times during the exploratory phase of this work, obtaining representative data remains a challenge. Comments from participants indicate that, before we can focus on the representativeness of the data, we must first get buy-in from national authorities. Presumably if the increase in AMR is felt more acutely in the years to come, national authorities are likely to agree to more active AMR surveillance, and to work with other countries to make sure the framework is representative. It was also emphasized that, in some countries, the role of private laboratories is so important that their participation will be necessary for representation, but they may be reluctant to provide services without clear financial benefits or other incentives^6^.

### AST Methods: Phenotypic vs. Genotypic Susceptibility

In most public AMR surveillance systems, phenotypic resistance is the basis of AST reporting, and genotypic susceptibility is optional and limited to a number of common resistance genes that are easy to test for, like extended-spectrum beta-lactamases (ESBL) in Gram-negative pathogens or mecA resistance genes in *Staphylococcus aureus*.

Phenotypic resistance is determined from the concentration of the antibiotic that inhibits bacterial replication *in vitro* and is used to predict whether or not a drug will be capable of inhibiting the bacteria *in vivo*, and thus improve clinical outcome. Genotypic resistance is determined by the presence of specific genes or plasmids that are known to confer decreased drug susceptibility. Presence of resistance elements does not always have direct treatment implications, as the bacterium may not express its resistance, or only express low-level resistance.

While genotypic analysis has been an integral part of some industry-sponsored AMR surveillance systems (e.g., SENTRY and PROTEKT), it should not be considered essential to the basic surveillance framework for antibiotics. The focus of the laboratories reporting AST to participating hospitals should be on phenotypic resistance, which can detect emerging resistance and is important to inform prescribing. However, where possible, new resistant isolates should be retained for genotypic analysis and re-analysis with new analytical techniques to understand the mechanism of action, pattern of spread (stand-alone mutations, clonal spread, or horizontal transfer of resistance genes) and inform diagnostic tests at the local level and for reference laboratories (13–15). At the level of reference laboratories, it is important to use both phenotypic and genotypic tests, as the latter can detect the genetic resistance mechanism (e.g., beta-lactamase production) needed for molecular epidemiology and development of reliable gene-based, diagnostic tests.

### Type of Sampling: Representativeness, Population, Isolate Type, and De-Duplication Strategy

In a perfect world, AMR surveillance for a new antibiotic would capture drug-specific AST data of all pathogens causing infections (or even colonization) within a population. Second best is to capture a sample with enough external validity to draw conclusions regarding the general level of resistance to the new antibiotic within the population. However, resources are limited, and sacrifices around representativeness have to be made. This may mean focussing on a patient population in which resistance is most likely to emerge. For example, surveillance efforts could be focused on hospitals where the new drug is being used extensively. Experts agreed that national authorities are best placed to decide when to proceed to more representative sampling by including routine AST data from hospitals that don't use the drug (transition to more routine AST is discussed below).

Participants in this study agreed that isolates from all age groups, including children, should be included. However, a newly approved novel drug is unlikely to have received regulatory approval for use in children, so any resistance emergence in this age group would be associated with transmission or off-label use.

Most respondents felt that all clinical samples should be tested for the novel drug and reported to the surveillance system. If resources are limited, a predetermined number of samples per hospital should be used. However, predetermined numbers need to be based on levels of perceived prevalence, something not possible for a new drug, such that an anticipated level of prevalence would have to be used.

In Delphi 1, the specimen type (i.e., blood, urine, sputum, wound, broncho-alveolar lavage) that should be covered was not yet discussed, as it was assumed that this would be highly dependent on the specific new treatment; pathogen coverage, indication etc. Therefore, this was addressed in the “super-penem” case study in Delphi 2, described below. The same holds true for pathogen type.

In routine surveillance, deduplication is needed in order to avoid biased results due to over-representation of individuals undergoing repeated sampling. Often the first isolate per person, per pathogen, per specimen type, per year is used, but this may not contain the most resistant strain. Opinion suggested that, in order to better capture emerging resistance, it may be useful to include, for each patient, the isolate with the most resistant profile, preferring the isolate with resistance to the new antibiotic.

### Data Quality: External Quality Control

To ensure coherent microbiological findings, laboratory services require regular quality control. A customized, surveillance-specific international scheme was considered optimal for new antibiotics.

### Desired Patient- or Hospital-Level Data

Efforts should be put toward collecting data on key indicators that can enhance our ability to understand and analyse the measured resistance levels to the new antibiotic. This could include aspects surrounding the patient, treatment, and setting for all resistant isolates. In the case of limited resources, the consensus order of importance, from most to least important, is: laboratory quality, clinical outcome data, individual risk factor data, and infection and prevention indicators (i.e., hand hygiene compliance).

### Reporting: From Proportion to Incidence

For routine surveillance, the level of AMR can be expressed in different ways, depending on the denominator used. The most common indicator is the proportion of resistance amongst all samples positive for a specific pathogen, followed by prevalence (denominator = patients) and incidence (denominator = patient-days at risk). The objective of the surveillance system should inform the most appropriate indicator(s). Table 2 lays out the most important advantages and disadvantages of the different AMR surveillance metrics. All of the reported measures can be influenced by ascertainment bias, especially in settings with very low, routine sampling rates, but the impact will be different. In most cases, low sampling rates are associated with selectively sampling those patients that are most likely to be infected by a drug-resistant pathogen, for example after empirical treatment failure. This means that the number of susceptible infections is more heavily underreported than the number of resistant infections. In this scenario, proportions will overestimate resistance proportions, while prevalence and incidence will underestimate the burden of resistance. This is due to the numerator (slightly underreported) and denominator (more strongly underreported) of proportions being affected by sampling bias, while for prevalence and incidence only the numerator is influenced.

**Table 2:** Advantages and disadvantages of different expressions of resistance (in routine surveillance).

	**Pro**	**Con**
Resistance proportion (lab based)	Relatively easy to measure, only laboratory data is required Useful for local treatment decisions	Changes in the prevalence of the susceptible bacterial population will influence the proportion (e.g., due to antibiotic use)Increasing proportions do not necessarily reflect an increase in the absolute number of resistant isolatesSelective sampling can easily result in an overestimate of resistance levels
Prevalence (patient based)	It indicates the absolute size of the problem It can be used to estimate the burden of disease assuming an appropriate level of routine sampling	Infections of short duration will be underrepresentedIt will only provide a snapshot of the situation at a specific point in time
Incidence (patient-days at risk)	It indicates the risk of acquiring a resistant infection It can be used to estimate the burden of disease assuming an appropriate level of routine sampling Proper picture of disease occurrence over a longer period of time	It requires appropriate de-duplication to prevent double countingRequires combining laboratory and hospital information

For surveillance of resistance emergence to newer drugs, the expert consultation echoed the idea that normalization of the resistance data (using a denominator) may not be necessary. Having the number of isolates and patients is sufficient in Phase 1. However, as surveillance transitions to being more routine in Phase 2, normalization of the data becomes increasingly useful.

### Reporting: Frequency

The higher the reporting frequency the better, especially for early warning systems and for new antibiotics needed in case of limited treatment options, but this, of course, depends on the level of available resources. An automated surveillance network would allow for real-time reporting, which would be ideal in this setting. All participants agreed this would be the way forward, but would not be feasible right now.

### Reporting: Destination of Reports

The majority of respondents agreed that the resistance surveillance data should be available to a wide array of users, including the public. The EARS-Net model is thought to be a workable one as anyone in the world can query the aggregated, anonymised data. Detailed, case-based data (without confidential details) are also available for distribution following a request, justification review, and approval/denial process for requests from non-participating countries. Data requests from participating countries, made through participating centers, are always approved. It should be noted that, legally, ECDC is obliged to give out any data that is collected to any EU citizen based on request. When asked if the EARS-Net data access policy should be adapted in any way for a surveillance system for newer antibiotics, participants felt that a policy like the one outlined here would be sufficient.

### Type of Sampling: One Health

Whilst this exercise focussed on human health, it did also explore views on the necessity of extending surveillance of new antibiotics to other sectors. Half of the respondents indicated that a One Health approach is paramount, as resistance will spread in all environments, and treatment of animals with new antibiotics should not be neglected (especially outside Europe and the United States). Although the One Health perspective is important, the ability to extend the surveillance system to animals (pets, food-production) and the environment (production effluent, wastewater) doesn't seem entirely feasible straight away, as it depends on the availability of resources and infrastructure. Therefore, we suggest that the enhanced surveillance framework should first focus on humans and be extended to other sectors only when greater resources become available. Also, as stressed by Grundmann et al. (14), care should be taken not to overburden the capacities of references laboratories in asking them to handle samples from food, water and veterinary sources.

### The “Super-Penem” Case Study

In order to explore nuanced preferences on issues with numerous dimensions, the group was asked to consider the case of a fictional new super-penem, which is an antibiotic active against carbapenemase-producing Enterobacteriaceae (CPE), MDR *Pseudomonas* species and MDR *Acinetobacter* species, but is not active against resistant Gram-positive pathogens, and has been approved for complicated urinary tract infection (cUTI) in adults.

*Transitioning from Phase 1 (Early warning) to Phase 2 (Routine surveillance)*. In order to explore the level of resistance at which the transition from Phase 1 and Phase 2 should take place, participants were given the classification from Grundmann and the CNSE Working Group, provided in box 1.

Box 1 **Timing of transition from early warning surveillance to routine surveillance along the epidemiological timeline (Stages defined by Grundman and the CNSE Working Group 2010).**

- Stage 0: No cases of resistant infections reported
- Stage1: Sporadic occurrence (Single cases, epidemiologically unrelated)
- Stage 2a: Single hospital outbreak (Outbreak defined as two or more epidemiologically related cases in a single institution)
- Stage2b: Sporadic hospital outbreaks (Unrelated hospital outbreaks with independent, i.e., epidemiologically unrelated introduction or different strains, no autochthonous interinstitutional transmission reported)

**Transition from Phase 1 (Early warning) to Phase 2 (Routine surveillance)**

- Stage 3: Regional spread (More than one epidemiologically related outbreak confined to hospitals that are part of a regional referral network, suggestive of regional autochthonous interinstitutional transmission)
- Stage 4: Inter-regional spread (Multiple epidemiologically related outbreaks occurring in different health districts, suggesting inter-regional autochthonous inter-institutional transmission)
- Stage 5: Endemic situation (Most hospitals in a country are repeatedly seeing cases admitted from autochthonous sources)

Participants agreed (*N* = 8/10) with the suggestion that the transition would be appropriate between Stage 2b and Stage 3. One dissenting voice suggested that such a super-penem would be of such importance in treating the pathogens listed, that the transition should take place earlier, after sporadic detection, i.e., after Stage 1. A second dissenting voice stressed that, in a country with high endemic carbapenem resistance, the transition should be after Stage 1. These responses highlight the importance of availability of alternative treatments in determining when the transition should take place: the fewer the treatment alternatives, the more closely we need to follow resistance. These considerations echo the reasoning of Cornaglia and ESGARS colleagues (16)**: “**If surveillance detects resistance in a dangerous organism, with no or few alternative drugs capable of controlling it, even a very low resistance rate should be considered high risk, and appropriate action should be planned.”

In this specific setting, it was expected that urine samples would be the most logical specimen of interest to report AST results to the enhanced surveillance system. Indeed, all participants agreed that urine samples were essential. In addition, they mentioned blood as essential, which is not surprising as urosepsis is an important complication of cUTI. Three out of nine thought that sputum and wound samples were also very important to include, to detect resistance emergence as early as possible. This clearly indicates that, for enhanced surveillance for new antibiotics, there will be no “one size fits all” solution with regards to specimen type.

*Isolate type*. Participants were asked to rank five suggestions about what isolate type should be collected according to perceived appropriateness, with the assumptions that resources are not a major constraint; that any type of sample is available—BSI, UTI, etc.; and that MDR signifies resistance to two or more antibiotic classes.

The first ranked response (average score 4.3/5) was “*All CPE isolates, regardless of whether the patient was treated with the drug*,” suggesting a strong emphasis on keeping difficult-to-treat infections treatable and keeping track of any emergence or transmission.
The second ranked response was “*Isolates from all patients who are infected with a MDR pathogen and who received the drug*” (score 3.9). This places emphasis on (added) resistance emergence within any already harder-to-treat infection, and co-lateral damage to the microbiome.
The third, “*All MDR Escherichia coli isolates, regardless of whether the patient was treated with the drug*” (score 3.1) suggests an emphasis on (added) resistance emergence within any already harder-to-treat infection. This result also emphasizes the importance of keeping that specific infection treatable and keeping track of emergence and transmission. The greater number of isolates in this case translates into higher probabilities of finding resistance emergence.
Lower ranked was “*All E. coli isolates, regardless of whether the patient was treated with the drug*” (score 2.5), which suggests that casting the figurative net as wide as possible to detect any resistance emergence to the new drug should not be a main priority.
The lowest rank was given to “*Isolates of all bacteria that could be co-presenting in the patient treated with the drug, even those bacteria not causing the primary infection*” (score 1.2), which reflects a lower priority given to potential co-lateral damage.

This questioning explored where priorities laid between two very different strategies: aiming to achieve a high probability of discovering new, emerging resistance through large sample size on one end, and maximizing clinical impact by focussing on the emergence of untreatable MDR infections, based on the indication of the drug, on the other end. The expert group placed the fulcrum on the side of clinical impact maximization.

### Limitations

One important limitation in this study is that it included 10 experts, who were all based in high-resource settings, which both narrows the set of possible viewpoints it gathered as well as the external applicability of its findings. While the findings can apply to surveillance systems in any part of the world, this study does not address fundamental challenges in implementing wider resistance surveillance where there are broader systemic challenges and very limited resources. The study also did not address the possible enhancement of surveillance through novel technologies. While we examined the level of priority associated with LIS connectivity and reporting automation (which, for most surveillance systems, would represent a substantial technological advance), we did not explore the potential use of any other new technology likely to have an impact on our ability to collect data and report on antimicrobial resistance [e.g., “smart” antibiograms (47)].

## Conclusion

AMR surveillance plays an essential role in our ability to preserve effective antibiotics, through early detection of resistance emergence and spread, followed by adequate intervention strategies. The slow pace of novel antibiotic development, combined with emergence of difficult-to-treat infections, makes our understanding of all aspects of emerging resistance to newer antibiotics ever more pressing. Yet current surveillance systems do not collect or report AST data for newer antibiotics, and are not designed to do so, as they would need to match all design issues described in this article. The critical issues that will need to be considered in designing an enhanced surveillance system that includes newer, last resort drugs can be summarized as follows in this call for action above in Box 2.

Box 2 **Call for surveillance for early detection of resistance to new antibiotics**

Early resistance surveillance should begin as soon as a new antibiotic is available for treatment, and the system should transition to more routine surveillance once sporadic, unrelated hospital outbreaks have occurred. National authorities should remain in charge of surveillance activities, but with coordination provided by a more centralized authority. As political support for resistance surveillance increases within individual countries, and adequate funding follows, the representativeness and overall quality of data contributed by national health authorities should be held to a higher standard. Financial contribution (from the central governing body) to support data collection activities in individual countries is important to improve the ability of the governing body to make requests to national authorities in the name of greater harmonization and representativeness—which improves our ability to respond on a regional level.

A well-funded surveillance scheme can of course be more ambitious and should include a capacity building element to continuously improve the system internally (increase expertise, improve data quality, increase efficiency) and expand the network geographically and over time. Finally, in extending resistance surveillance to newer antibiotics we should focus our efforts on identifying resistance that occurs in already difficult-to-treat infections, in particular, such that we can respond in time to contain transmission of infections that are completely untreatable. The increasing threat of AMR, combined with the paucity of novel treatment strategies becoming available, requires an adequate public health response, which should include a bespoke AMR surveillance strategy for antibiotics coming to the market today.

## Data Availability Statement

The original contributions generated for this study are included in the article/Supplementary Material, further inquiries can be directed to the corresponding author/s.

## Author Contributions

CM and MK reviewed the literature, developed the surveys with close support from SH, and analyzed responses. CM, MK, and SH identified the first potential interviewees. CM conducted the interviews to narrow down the list of crucial issues and identify additional interviewees and wrote the manuscript with close support from MK and SH. Members of the Enhanced surveillance expert consensus group provided input through two survey rounds and provided input into the interpretation of findings and conception of the manuscript. MK developed the table comparing the choice of denominator. All authors contributed to the article and approved the submitted version.

## Conflict of Interest

The authors declare that the research was conducted in the absence of any commercial or financial relationships that could be construed as a potential conflict of interest.

## Footnotes

^1^*Escherichia coli, Klebsiella pneumoniae, Pseudomonas aeruginosa, Acinetobacter species, Streptococcus pneumoniae, Staphylococcus aureus, Enterococcus faecalis, Enterococcus faecium* (1).

^2^EWRS and EPIS.

^3^Geographic origin was not an established parameter. The inclusion of individuals from Europe and the United States was a result of the selection methodology.

^4^The 11 individuals identified (10 of whom participated) were all of those deemed to have a very deep understanding of AMR. The individual areas of personal expertise varied amongst several disciplines. It was deemed more important to acquire high quality input than to achieve a greater number of responses.

^5^In some cases, university laboratories can serve this purpose.

^6^In some settings, private laboratories are willing to join public networks without financial remuneration in order to receive more training (to support quality assurance), to partake in research, to boost prestige, or simply to support public health.

## CANSORT-SCI Affiliations

- Arjun Srinivasan. Division of Healthcare Quality Promotion, Centers for Disease Control and Prevention, Atlanta, GA, United States.
- Benjamin J. Park, International Infection Control Program, Division of Healthcare Quality Promotion, Centers for Disease Control and Prevention, Atlanta, GA, United States.
- Diamantis Plachouras. European Centre for Disease Prevention and Control, Solna, Sweden.
- Evelina Tacconelli. Infectious Diseases Section, Department of Diagnostics and Public Health, University of Verona, Verona, Italy.
- Jean Patel. Beckman Coulter, Sacramento, CA, United States.
- John Stelling. Brigham and Women's Hospital, Microbiology Laboratory, Boston, MA, United States.
- Katie Hopkins. Public Health England, Healthcare Associated Infections & Antimicrobial Resistance Division, National Infection Service, London, United Kingdom.
- Ole Heuer. European Centre for Disease Prevention and Control, Solna, Sweden.
- Petra Gastmeier. Institute of Hygiene and Environmental Medicine, Charité Universitätsmedizin Berlin, Berlin, Germany.

## Supplementary Material

Appendix 1 Delphi survey 1.

Appendix 2 Delphi survey 2.

Appendix 3 Surveillance design issues identified in the mapping exercise.
