# Supplementary material for: Surveillance of Resistance to New Antibiotics in an Era of Limited Treatment Options
Source: Front Med (Lausanne). 2021 Apr 19;8:652638. doi: 10.3389/fmed.2021.652638 (PMC8091962; doi:10.3389/fmed.2021.652638)
Supplement: Appendix 1 — Delphi survey 1. [file Data_Sheet_1.PDF]

Surveillance of resistance to recently launched antibiotics for Gram-negative infection: moving towards greater agreement on the optimal organization, sampling frame, reporting, outputs, and definitions.

Note, this study explores the optimal features of a (potential) surveillance framework for antibiotics that have received market approval (e.g. by EMA, FDA) and are available on the market but that have not yet been incorporated within the main surveillance networks such as EARS-Net and GLASS.

Why improve surveillance of emerging resistance to new antibiotics?

Faced with a decreasing selection of therapeutic options for some Gram-negative infections and the lack of true novelty amongst new antibiotics public health authorities should consider undertaking resistance surveillance of new antibiotics early on. Early detection of emerging resistance is arguably increasingly important in order for us to:

- **quantify prevalence and act sufficiently quickly with regard to prevention & control measures**
- **adapt lab procedures (including procuring appropriate technologies)**
- **switch to more appropriate treatments**
- **build and adapt monitoring capacity**
- **steer R&D towards the development of new drugs that can take over treatment of the relevant infections**
- **examine the underlying epidemiology and the role of antibiotic consumption within it**

Currently surveillance of resistance to new antibiotics by the public sector is extremely limited, with almost all regular surveillance activities initiated by the private sector. Information from these surveillance activities remain the property of companies themselves, intended to support applications for (additional) market approval or to satisfy post-launch requirements to the regulator. A selection of findings from these surveillance activities are sometimes published but with little detail surrounding the sampling protocol, making it difficult to compare with other findings, and difficult to assess the level of representativeness and overall usefulness for public health policy.

This work explores how we might move towards an inter-connected, standardized,

representative, publicly-led system for monitoring phenotypic resistance to newly launched drugs.

Resistance surveillance for new antibiotics

About this exercise

This questionnaire is sent to you as Part 1 of a modified Delphi exercise intended to gather responses from a handful of experts. At the end of this round of questioning responses will be consolidated and any points of disagreement will be identified. In a few weeks respondents will be sent the consolidated, more detailed version for approval or potential further comment as Part 2 of this process (the hope is that we can move towards some sense of consensus). Aggregated (anonymized) results of the exercise will be published. All participants will be invited as authors.

Practical information

**While each question includes possible responses, additional responses and/or additional detail may be included on the lines provided at the bottom of each question.**
**If possible, please complete the questionnaire in one sitting. If this is not possible you can finalize it at a later time as long as you are using the same computer and the same browser -- Google Chrome, Internet Explorer, Firefox -- and you don't empty the navigation history (don't empty the cookies).**
**If you have any questions please get in touch with Dr Chantal Morel.<br>Email chantal.morel@unige.ch or phone +41766091502**
Thanks for your participation! Chantal Morel, Marlieke de Kraker, and Stephan Harbarth

* 1. Contact information

Name ____________________

Resistance surveillance for new antibiotics

Getting started

* 2. When should resistance surveillance for newly launched antibiotics begin?

- ○ When a minimal level of use is reached
- ○ When the first resistant isolates are detected
- ○ When BOTH minimal level of use is reached and the first resistant isolates are detected
- ○ Other (please specify)

Resistance surveillance for new antibiotics

Getting started

* 3. What system should we rely on for detecting the first resistant isolates?

(multiple answers possible)

- ☐ Agreements with existing reference labs
- ☐ Agreements with existing (largely private) surveillance networks
- ☐ WHO early warning system: EAR. Note, this assumes that newly launched antibiotics would be added to the EAR framework.
- ☐ Other (please specify in the Comments box)

Comments

Resistance surveillance for new antibiotics

Getting started

* 4. How should we determine the minimal level of use necessary to begin surveillance?

(multiple answers possible)

- [ ] Company-provided sales data
- [ ] Public purchaser data
- [ ] Presence of the drug on national prescribing guidelines
- [ ] Hospital pharmacy purchasing data
- [ ] Other (please specify in the Comments box)

Comments

Resistance surveillance for new antibiotics

Sampling frame

* 5. What type of institutions should be reporting AST results to the system?

(please select ALL that apply)

- [ ] Public sector hospitals (all)
- [ ] Secondary care centres (all)
- [ ] Tertiary care centres (all)
- [ ] Specialized care hospitals (all)
- [ ] Selected public sector hospitals (sentinel system)
- [ ] Private and public sector hospitals (all)
- [ ] One single, national reference lab
- [ ] Other (please specify in the Comments box)

Comments

* 6. From whom should samples be collected for this surveillance? (weighing resources against potential for bias)

- ◯ All patients with positive clinical cultures for any type of Gram-negative bacteria whose sample undergo AST
- ◯ All patients with positive clinical cultures for specified MDR-GNB (e.g. CPE)
- ◯ All patients with microbiological samples for whom current standard treatment do not work, and who would qualify for new drug
- ◯ Patients with specified risk factors (please specify in the Comments box)

Comments

* 7. How should the samples (from patient population defined earlier) be chosen within this selection process?

- ◯ All hospital-derived samples
- ◯ All samples from selected wards
- ◯ A predetermined number of samples per hospital
- ◯ A predetermined number of samples per (selected) ward
- ◯ Other (please specify in the Comments box)

Comments

Resistance surveillance for new antibiotics

Sampling frame

* 8. At what level should we perform a random selection? (instead of including all of them)

(multiple answers possible)

- ☐ selection of hospitals
- ☐ selection of wards
- ☐ selection of samples
- ☐ Comments (please specify)

* 9. What types of samples should be included?

- ◯ Only clinical cultures
- ◯ Clinical cultures and routine screening swabs to detect asymptomatic carriage for infection control purposes

Comments

* 10. Samples should include which pathogens? (weighing resources against potential for bias)

- ◯ All those relevant for the indication of the new antibiotic
- ◯ All Gram-negatives irrespective of the antibiotic susceptibility
- ◯ All multidrug-resistant Gram-negatives from the WHO Pathogen Priority List?
- ◯ All pathogens from the GLASS framework (*Acinetobacter* spp., *Escherichia coli*, *Klebsiella pneumoniae*, *Neisseria gonorrhoeae*, *Salmonella* spp., *Shigella* spp., *Staphylococcus aureus*, and *Streptococcus pneumoniae*)
- ◯ Only 1 specific bug-drug combination as key indicator
- ◯ A selected list of pathogens (please specify in Comments box)

Comments

* 11. Appropriate age group of patients?

- ◯ All patients, including children
- ◯ Adults only (18+)

Comments

Resistance surveillance for new antibiotics

Sampling frame

Choice of denominator

AMR can be expressed in different ways, depending on the denominator used.

- **Proportion of resistance is the indicator most commonly used, where the numerator is the total number of resistant clinical isolates, and the denominator is the total number of clinical isolates (of the same body site(s) and species).**
- **Prevalence is the number of patients with a resistant isolate over the total number of patients tested at a certain point in time.**
- **Incidence uses the same numerator, but the denominator has a time component in it, different denominators can be used, including patient-days, hospital admissions, beds etc.**

The objective of the surveillance system will in most cases inform the most appropriate indicator(s). In the table below you will find the most important pros and cons of the different measures.

	**Pro**	**Con**
**Resistance proportion**	• Relatively easy to measure, only laboratory data is required<br>• Useful for local treatment decisions	• Changes in the prevalence of the susceptible bacterial population will influence the proportion (e.g. due to antibiotic use)<br>• Increasing proportions do not necessarily reflect an increase in the absolute number of resistant isolates<br>• Selective sampling can easily result in an overestimate of resistance levels
**Prevalence**	• It indicates the absolute size of the problem<br>• It can be used to estimate the burden of disease<br>• Less sensitive to biased sampling practices than proportions	• Infections of short duration will be underrepresented<br>• It will only provide a snapshot of the situation at a specific point in time
**Incidence (general)**	• It indicates the risk of acquiring a resistant infection<br>• It can be used to estimate the burden of disease<br>• Proper picture of disease occurrence over a longer period of time<br>• Less sensitive to biased sampling practices than proportions	• It should exclude patients not at risk for the specific condition due to immunity for example<br>• It requires appropriate de-duplication to prevent double counting<br>• Requires combining laboratory and hospital information
**Incidence, per 1000 patient-days**	• Takes into account occupancy	• Does not account for turn-over rates, i.e. hospitals with different discharge policies may not be comparable
**Incidence, per 100 beds per time unit**	• Easy to measure	• Does not account for turn-over rates<br>• Does not account for occupancy, i.e. occupancy changing over time or between hospitals will reduce comparability
**Incidence, per 100 admissions per time unit**	• Takes into account turnover rate	• Does not account for occupancy

* 12. Assuming some level of acquired resistance, what denominator(s) should be used?

(multiple answers possible)

- [ ] Isolates (proportion: x number of resistant isolates / total number of selected isolates)
- [ ] Patients (prevalence: e.g. x number of positive patients / total number of selected patients)
- [ ] Patient-days (incidence: e.g. x number of MRSA per 1000 patient-days)
- [ ] Patient-beds (incidence: e.g. x number of MRSA per 100 beds per time unit)
- [ ] Patient admission (incidence: e.g. x number of MRSA per 100 admissions per time unit)
- [ ] Other (please specify in Comments box)

Comments

* 13. What indicators would be useful to assess the potential for detection bias within the provided data?

(multiple answers possible)

- [ ] Number of blood cultures conducted per 1000 patient-days overall within the contributing hospital
- [ ] Number of positive blood cultures over total number of blood cultures overall within the contributing hospital
- [ ] Number of other (e.g. urine, screening, etc.) cultures per patient-days. (Please specify in Comments box)

Comments

Resistance surveillance for new antibiotics

Organization

* 14. Where should the funding for this surveillance come from?

(multiple answers possible)

- [ ] Public funding (national level)
- [ ] Public funding (EU level)
- [ ] GLASS (via WHO)
- [ ] EARS-Net (via EU)
- [ ] Mix of public and private funding
- [ ] An association of pharmaceutical companies (e.g. EFPIA)
- [ ] An association of pharmaceutical companies that have a newly-launched antibiotic
- [ ] Other (please specify in Comments box)

Comments

* 15. Who should be in charge of governance?

- ○ A health authority from one country
- ○ An independent, new international health authority combining representation from the full group of participating countries
- ○ An existing international health authority combining representation from the full group of participating countries (e.g. a new arm of an existing organization such as ECDC, WHO, etc.)
- ○ A special NGO dedicated to AMR
- ○ An independent public-private partnership
- ○ A large, globally-oriented, not-for-profit organization (e.g. Wellcome Trust)
- ○ An association of pharmaceutical companies
- ○ Other (please specify in Comments box)

Comments

* 16. How should the health system interact with the surveillance body?

- ○ Direct contact between labs and the surveillance body
- ○ Pooling of data at national/sub-national level first and then fed into the surveillance body
- ○ Other (please specify in Comments box)

Comments

* 17. In addition to the surveillance body, which institutions should have access to the complete, disaggregated (anonymized with respect to patient identity and hospital name) data set?

(multiple answers possible)

- [ ] All participating hospitals and laboratories
- [ ] All governments/health authorities of participating countries
- [ ] All governments/health authorities of non-participating countries
- [ ] The public
- [ ] Other parties (please specify in Comments box)

Comments

* 18. Should the surveillance body collect the physical isolate in order to facilitate, for example, full exploration of the underlying resistance mechanisms (now and in future), capacity-building purposes, etc.?

- ( ) Yes
- ( ) No

Comments

Resistance surveillance for new antibiotics

Organization

* 19. What should be the frequency of data submission given potential uses of the data (e.g. resistance trends analysis to inform policy-making, treatment decisions/ABS, alerting outbreaks, etc.)?

- ( ) Yearly
- ( ) Quarterly
- ( ) Monthly
- ( ) Daily/real time

Comments

* 20. Which external quality control system should be used?

- ◯ Local scheme
- ◯ Standardized international scheme
- ◯ Customized, surveillance-specific international scheme
- ◯ Other (please specify in Comments box)

Comments

* 21. How important is it to collect these additional data from hospitals/labs? (score 1 to 5)

	1. Not at all important	2.	3.	4.	5. Extremely important
Infection and prevention indicators	◯	◯	◯	◯	◯
Risk factors	◯	◯	◯	◯	◯
Clinical outcome	◯	◯	◯	◯	◯
Indicators for lab quality	◯	◯	◯	◯	◯

Comments

* 22. As we are considering the surveillance of resistance to antibiotics that have become available relatively recently and will be kept for human use only we have assumed that sampling from the environment and from veterinary settings would not be essential for this endeavour. Do you agree with this assumption?

- ◯ Yes
- ◯ No (please explain)

* 23. What are the important outputs the surveillance system can offer back to participating entities?

(multiple answers possible)

- [ ] Warning of outbreak and early emergence data to steer infection control purposes
- [ ] Benchmarks -- figures showing how their data compares to others
- [ ] Informative and elegantly-presented summary reports
- [ ] Other (please specify)

* 24. When should we end these surveillance activities?

- ○ If resistance remains below a very low threshold
- ○ If resistance surpasses a very high threshold
- ○ After a predetermined period of time has elapsed
- ○ After 'regular' (e.g. EARS-Net) surveillance has taken over?
- ○ Other (please specify)

25. Are there any other key features to make the results of this surveillance system more accurate, representative, standardized, or otherwise more acceptable to stakeholders?

1.

2.

3.

4.
