# Supplementary material for: Surveillance of Resistance to New Antibiotics in an Era of Limited Treatment Options
Source: Front Med (Lausanne). 2021 Apr 19;8:652638. doi: 10.3389/fmed.2021.652638 (PMC8091962; doi:10.3389/fmed.2021.652638)
Supplement: Appendix 2 — Delphi survey 2. [file Data_Sheet_2.PDF]

Welcome to the second round of this Delphi exercise.

Thank you again for having participated in the first round. We received much useful information and found numerous areas of agreement surrounding the desired design features for a potential resistance surveillance framework for newer antibiotics.

Findings from Round 1 are summarized below and a more detailed version will be included in the paper that you will all be invited to be part of.

Findings from Round 1

***General findings*: The group see a clear need for a system to detect/report resistance to newer antibiotics. The system should be decentralized, run largely from national level but coordinated by an arm of an existing international public institution (e.g. ECDC, WHO).**

Surveillance of newer antibiotics should be considered in two phases:
1. Early warning surveillance to detect emergence of resistance, followed by
2. (Enhanced) Routine surveillance

***Early warning surveillance*. The majority of respondents expressed that ideally early warning resistance surveillance should begin as soon as a novel antibiotic is launched onto the market, regardless of the level of use. Ideally, all novel antibiotics should have very low levels of use but this should not preclude surveillance. So as soon as a new antibiotic is introduced in a hospital, it makes sense to begin susceptibility testing and surveillance locally.**

Immediate early warning surveillance was deemed to be particularly important for drugs that are critically important, such as for the treatment of infections with limited treatment options (e.g. MDR) and priority AMR threats (e.g. CRE). Such immediate surveillance was less important for new members of existing classes that do not really add to the existing treatment options.

Half of respondents felt that for initial reporting of resistance emergence we could rely on agreements with existing reference labs while the other half felt we should rely on agreements with existing (largely private) surveillance networks to report resistance proportions to the surveillance governing body. All hospitals would be assumed to send MDR isolates to a reference lab where resistance to the novel drug could be confirmed. Many respondents felt that the WHO early warning system (EAR) – a reporting system (not a surveillance system) -- could also be useful for reporting initial resistance emergence, assuming subsequent verification.

Denominator information was not seen to be essential for early warning surveillance -- resistance proportions were seen to suffice.

Resistance surveillance for newer antibiotics

Summary of findings from Round 1

***Routine surveillance*. As resistance becomes more prominent more routine surveillance will need to be enhanced. Generally speaking the selection of institutions for routine surveillance should facilitate as good coverage as possible and, ideally, reporting of AST results of new antibiotics should be done for all hospital-derived samples, including pediatric isolates, by all parties involved in monitoring activities. But, practically speaking, this depends on the level of available resources and infrastructure. For example, if it is possible to connect all LIS to a central database then all available AST results can be automatically uploaded to the surveillance structure.**

***Funding*. National public sector was deemed to be an appropriate source of funding by all respondents, followed by public funding from EU level and a mix of public and private funding (the latter two having achieved the same level of support). Two respondents stressed that ideally, we'd get a pool of funds with contributions from many sources. For example, from the private sector part of the licensing fees for a new antibiotic could be put towards this cause. However, a mix of private and public funds could be difficult as public agencies are often not allowed to accept private funding.**

***Governance*. By far the most popular response was that an existing international health authority combining representation from the full group of participating countries (e.g. a new arm of an existing organization such as ECDC, WHO, etc)**

should be in charge of governance.

The data should be pooled at national/sub-national level first and then fed into the surveillance body. Indeed the national level will normally not like AMR data from the country to be shared without national curation. First level of data collection, cleaning, validation, and confirmation at national level would be appropriate, especially from the quality perspective. EARS-Net is considered a well-functioning, tried and tested example. However, for the purposes of early warning direct contact between the labs and the surveillance body may be required.

***Access to the data collected*. The majority of respondents agree that the data should be available to a wide array of users, including the public. The EARS-Net model is thought to be a reasonable one. Anyone in the world can query the aggregated, anonimised data. Detailed, case-based data (without confidential details) are also available for distribution following a request, justification review, and approval/denial process. From participating countries, the reasoning for the data request does not have to be approved while from non-participating countries it does have to go for approval.**

Resistance surveillance for newer antibiotics

Summary of findings from Round 1

***Collection of the physical isolate*. Responses suggested that indeed there should be a structure in place to collect the isolate.**

***External quality control*. A customized, surveillance-specific international scheme was considered optimal for novel agents.**

***Reporting frequency*. The higher the frequency the better, especially for emerging resistance, but of course it depends on the level of available resources. A computerized network would allow for real-time reporting, which would be the way forward. Frequency of reporting is more important for drugs needed to treat MDR infections and for supporting early warning systems.**

***One Health*. Half of the respondents indicated that a One health approach is the way forward, as cross resistance will occur, and animal exposure (especially outside**

Europe) is likely. While the One Health perspective is an absolute priority, the ability to extend the surveillance system to animals (veterinary, food-producing agriculture) and the environment (production effluent, wastewater) will depend on availability of resources and available infrastructure. If resources are limited then the framework will begin with humans only and extend to other sectors as greater resources become available and/or new technologies are able to facilitate our efforts.

Resistance surveillance for newer antibiotics

Welcome to Round 2

* 1. Contact information

Name [ ]

2. Regarding early warning surveillance: Half of respondents in Round 1 felt that for initial reporting of resistance emergence we could rely on agreements with existing reference labs while the other half felt we should rely on agreements with existing (largely private) surveillance networks to report resistance proportions to the surveillance governing body.

Upcoming regulatory reform suggests that the balance of private and public activities in surveillance may change in the years to come. Surveillance activities of specialised private sector surveillance companies are expected to decrease. (For those who would like an update don't hesitate to get in touch: chantal.morel@unige.ch)

For those who initially reported that we should rely on these specialised private sector surveillance companies to report resistance emergence: Do you feel that it will be sufficient to rely more on public reference labs for this purpose?

- ◯ Yes, we can rely more on public reference labs (please also tick 'yes' if you already gave a preference for relying on public reference labs for detecting resistance emergence in Round 1)
- ◯ No, public reference labs are inadequate for detecting resistance emergence

Comments

[ ]

3. How should the role of the reference lab in detecting first cases of resistance be characterized?

- ◯ Passive (the reference lab receives no instructions or obligations from the surveillance body)
- ◯ Active (the reference lab receives guidelines and some kind of obligation to report to the surveillance body)

Other/Comments

4. Consider the case of a new “superpenem”, which is active against CPE, MDR Pseudomonas and MDR Acinetobacter (and not active against resistant Gram positive pathogens) and has been approved for cUTI in adults.

At what level of resistance should countries consider transitioning from an early warning system to a routine surveillance system? Using the classification from Grundmann and the CNSE Working Group (2010) here-below, the transition seems appropriate between Stage 2b and Stage 3. Do you agree?

Stage 1: Sporadic occurence (Single cases, epidemiologically unrelated)
Stage 2a: Single hospital outbreak (Outbreak defined as two or more epidemiologically related cases in a single institution)
Stage 2b: Sporadic hospital outbreaks (Unrelated hospital outbreaks with independent, i.e. epidemiologically unrelated introduction or different strains, no autochthonous inter-institutional transmission reported)
Stage 3: Regional spread (More than one epidemiologically related outbreak confined to hospitals that are part of a regional referral network, suggestive of regional autochthonous inter-institutional transmission)
Stage 4: Inter-regional spread (Multiple epidemiologically related outbreaks occurring in different health districts, suggesting inter-regional autochthonous inter-institutional transmission)
Stage 5: Endemic situation (Most hospitals in a country are repeatedly seeing cases admitted from autochthonous sources)

(classification from Grundmann and the CNSE Working Group Euro Surveill 2010 Nov 18:15;48)

- ◯ Yes
- ◯ No
- ◯ Other

Comments

5. Who should be in charge of selecting the hospitals that will contribute to routine surveillance?

- ○ National authorities
- ○ The surveillance governing body

Comments

6. In order to be a full member of the surveillance network, should countries have to provide data from a representative set of hospitals/labs?

- ○ Yes
- ○ No
- ○ Other

Comments

7. Findings from Round 1 suggest that EARS-Net has a good data access policy. Anyone in the world can query the database. But the information provided is only in aggregate and doesn't identify facilities or patients. The underlying data (without confidential details) are also available for distribution following a request, justification review, and approval/denial process. From participating countries, the reasoning for the data request does not have to be approved while from non-participating countries it does have to go for approval.

Do you feel that this data access policy should be adapted in any way for a surveillance system for newer antibiotics?

- ○ This approach would work well as it is.
- ○ This approach would need to be adapted.

Suggestions for how the data access policy should be adapted / Comments

8. Consider the case of a new “superpenem”, which is active against CPE, MDR*Pseudomonas* and MDR *Acinetobacter* (and not active against resistant Gram positive pathogens) and has been approved forcUTI in adults. Under routine surveillance, which data should be collected?

Please rank these according to perceived appropriateness

Assumptions: resources are not an issue; any type of sample -- BSI, UTI, etc.;MDR = resistant to 2 or more classes

- [ ] Isolates from all patients who have an MDR pathogen and who receive the drug
- [ ] All E coli isolates, regardless of whether or not the patient was treated with the drug
- [ ] All MDR E coli isolates, regardless of whether or not the patient was treated with the drug
- [ ] All CPE isolates, regardless of whether or not the patient was treated with the drug
- [ ] Isolates of all bacteria that could be co-presenting in the patient treated with the drug (even those bacteria not causing the primary infection)

9. Consider again the case of a new “superpenem”, which is active against CPE, MDR Pseudomonas and MDR Acinetobacter (and not active against resistant Gram positive pathogens) and has been approved for cUTI. Which sample types should be collected? *(please check all that apply)*

- [ ] Urine
- [ ] Blood
- [ ] Sputum
- [ ] Wound
- [ ] Other/Comments

10. Any further comments surrounding the ideal features of early warning or routine surveillance of newer antibiotics?
