# Supplementary material for: Surveillance of Resistance to New Antibiotics in an Era of Limited Treatment Options
Source: Front Med (Lausanne). 2021 Apr 19;8:652638. doi: 10.3389/fmed.2021.652638 (PMC8091962; doi:10.3389/fmed.2021.652638)
Supplement: Appendix 3 — Surveillance design issues identified in the mapping exercise. [file Data_Sheet_3.docx]

## Appendix 3. Surveillance design issues identified in the mapping exercise

Focus and purpose of surveillance

Timing

Start of early warning surveillance for newer drugs

Start of antibiotic specific routine epidemiological surveillance

Timing of transition from early warning to routine surveillance

When to stop routine surveillance

Governance

Structure

Data / Isolate contributing entities

Integration / collaboration of lab networks

National versus international capacities

Data ownership/Data access policy

Type of surveillance (active / passive, comprehensive/sentinel)

Overall financial support

Financial incentives to participating units

Data dissemination structure/technology/coding standards

Criteria for uptake of new pathogens into surveillance networks

Criteria for termination of AMR surveillance for specified drugs or specified bug-drug combinations

Laboratory (reference or local) or hospital-based

AST data

Type of resistance (antibiotic classes)

Phenotypic or genotypic approach

Typing of plasmids/chromosomal resistance

AST methods

Disk diffusion/ E-test / Automated systems

Genetic characterization of the detected resistance

MIC versus interpretation reporting

Breakpoint guidelines

Local versus central testing

Type of sampling

Which sectors to include / One health

Sample specimen (blood, urine, etc.)

Isolate type (clinical / screening)

De-duplication strategy (first isolate per patient, most resistant isolate)

Representativeness

Age

Gender

Urban/rural

Hospital/community

Pathogen

Choice of pathogen(s) in relation to indication of new antibiotic

Only MDR or independent of other resistance features

Desired patient-level data

Age of patient

Gender of patient

Clinical outcome

Risk factors

Desired hospital-level data

Control and prevention measures in place

Standardization in sampling

Experience indicators

Data quality

Data quality indicators

Definitions used for duplicate removal

Feedback and confirmation

Laboratory internal quality control system

Laboratory external quality control system

Reporting

Aggregation level of data

Stratification of results by (to maximize usefulness of the results and efficiency of use)

Format of results to allow for aggregation/comparison without physical access to isolates

Destination of reports

Reporting frequency

Specific indicators to report (to maximize usefulness and comparability of results)

Co-resistance

Proportion of different resistance mechanisms

Number of outbreaks or clusters

Specimen

Number of cases v number of patients with resistant infection

Sampling methods

Prevalence / incidence / resistance proportions

Denominators

Measure of resistance to novel drug

Possible separate indicator for resistance to AB classes other than the one to which the novel drug belongs

Age

Gender

Prior treatment history

Type of unit

Consumption level (of antibiotic of interest)
